# Case report: A 56-year-old woman presenting with torsades de pointes and cardiac arrest associated with levosimendan administration and underlying congenital long QT syndrome type 1

**DOI:** 10.1016/j.heliyon.2024.e29300

**Published:** 2024-04-10

**Authors:** Fengyan Zha, Xing Li, Hui Yin, Di Huang, Yu Du, Chuzhi Zhou

**Affiliations:** a Department of Surgical Intensive Care Unit, Fuwai Hospital Chinese Academy of Medical Sciences, Shenzhen, SZ, China; b Department of Surgical Intensive Care Unit, Fuwai Hospital Chinese Academy of Medical Sciences, Beijing, BJ, China

**Keywords:** Torsades de pointes, Levosimendan, Long QT syndrome, Implantable cardioverter defibrillator, Case report

## Abstract

Torsades de Pointes (TdP) is a malignant polymorphic ventricular tachycardia with heart rate corrected QT interval (QTc) prolongation, which may be attributed to congenital and acquired factors. Although various acquired factors for TdP have been summarized, levosimendan administration in complex postoperative settings is relatively uncommon. Timely identification of potential causes and appropriate management may improve the outcome. Herein, we describe the postoperative case of a 56-year-old female with initial normal QTc who accepted the administration of levosimendan for heart failure, suffered TdP, cardiac arrest, and possible Takotsubo cardiomyopathy, further genetically confirmed as long QT syndrome type 1 (LQT1). The patient was successfully treated with magnesium sulfate, atenolol, and implantable cardioverter defibrillator implantation. There should be a careful evaluation of the at-risk populations and close monitoring of the electrocardiograms, particularly the QT interval, to reduce the risk of near-fatal arrhythmias during the use of levosimendan.

## 1 Introduction

TdP, a rare but potentially lethal polymorphic ventricular tachycardia, is characterized by wide QRS complexes of gradually varying amplitude that appear to ‘twist’ around the isoelectric baseline on the electrocardiogram [1]. It is widely known that TdP is associated with the prolongation of QTc which may be congenital or acquired in the clinical setting of electrolyte disturbances, medication, cardiac disease, cerebrovascular injury, thyroid dysfunction, hypothermia, inflammation, autoimmunity, and many other factors [[1], [2], [3]]. Certain drugs, like class III antiarrhythmic drugs, antidepressants, and antibiotics, may induce QTc prolongation and TdP [4].

Levosimendan is a calcium sensitizer and potassium channel opener widely used in clinics [5]. However, several studies have unmasked relevant adverse effects of levosimendan, including gastrointestinal side effects, hypotension, ventricular arrhythmia, atrial fibrillation, and deaths [[6], [7], [8]]. It is noted that the history of TdP is a contraindication for using levosimendan, besides significant mechanical obstructions affecting ventricular filling or outflow or both [9].

Here we describe a postoperative case, with an initial normal QTc, presented TdP and cardiac arrest after levosimendan administration, and further gene screening confirmed underlying congenital LQT1.

### 1.1 Case presentation

**Fig. 1:**
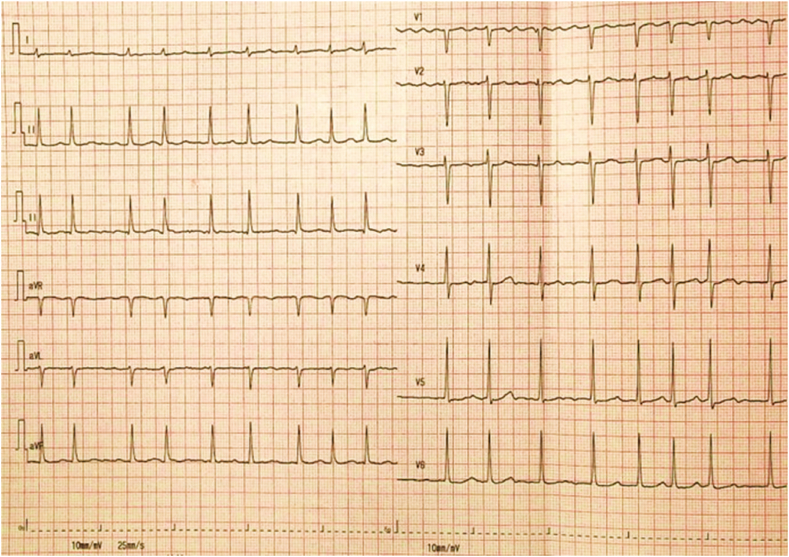
ECG on admission showing atrial fibrillation with a normal QTc interval of 435 ms under a heart rate of 93 bpm.

**Fig. 2:**
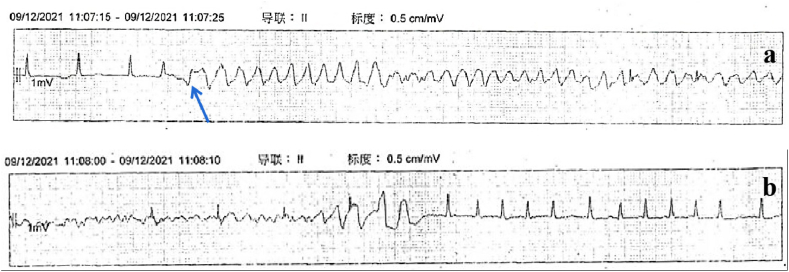
ECG monitoring showed an R-on-T event (arrow) that initiated the episode of TdP which deteriorated into ventricular fibrillation (a) and then converted to Af with a mild prolonged QTc of 454 ms under a heart rate of 140 beats per minute with compressions (b).

**Fig. 3:**
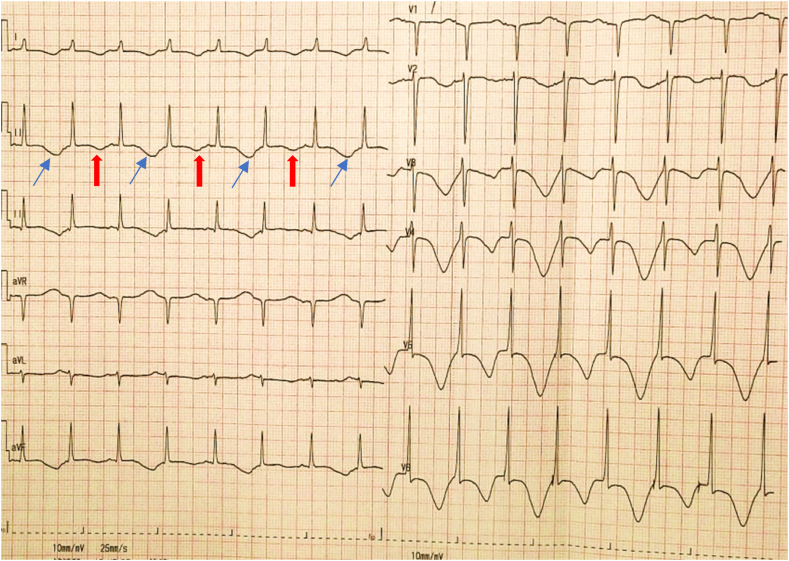
ECG showed T-wave alternation on 12 leads following the pattern of ABAB (arrow) on day 14.

**Fig. 4:**
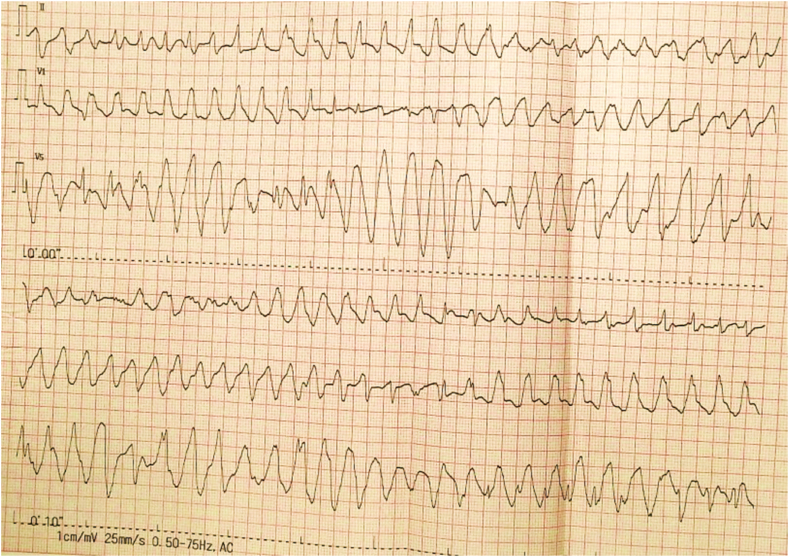
ECG monitoring showed episodes of TdP on day 14.

A 59-year-old female with a medical history of rheumatic valvular heart disease with atrial fibrillation (Af), hypertension, type II diabetes, and hyperlipemia presented to the cardiac surgery ward with chest tightness and palpitation for the past two months. She admitted a family history of the sudden death of her mother, but she had no history of syncope or TdP. She denied food and medication allergies. The patient was taking digoxin, furosemide, spironolactone, sacubitril valsartan sodium tablets, metformin, dapagliflozin, and atorvastatin at the time of presentation to the hospital. Physical examination showed a blood pressure of 115/89 mmHg, an irregular heart rate of 98/min with a pulse rate of 76/min, a respiratory rate of 16/min, oxygen saturation levels of 100 % at room air, a body temperature of 36.2 °C, and weight 61 kg. A systolic blowing murmur can be heard in the apical region. She had no rales on pulmonary auscultation. There was no pitting edema in the lower extremities. On admission, the N-terminal B-type brain natriuretic peptide precursor (NT-proBNP) was 2709 pg/ml (reference range 0–300 pg/ml). Autoantibody ANA and related antibodies were negative. Serum immunoglobulins were in the normal range. Interleukin-6 (IL-6) was 5.4 pg/ml (reference range 0–7pg/ml). The initial electrocardiography (ECG) showed Af with a normal QTc of 435 ms, as shown in Fig. 1. Transthoracic echocardiogram showed left heart enlargement, left atrium anteroposterior diameter of 54mm, left ventricular end-diastolic diameter (LVEDD) of 62mm, moderate-to-severe mitral regurgitation, mild-to-moderate tricuspid regurgitation and left ventricular ejection fraction (LVEF) of 57 %. After optimization of cardiac function and fluid status, the patient underwent mitral valve replacement, tricuspid valvuloplasty, COX-maze IV procedure, epicardial temporary pacing electrode implantation on day 8 of admission, and the postoperative ECG showed a normal QTc of 448 ms. On day 9, a bedside echocardiogram showed that LVEDD was 56mm and a reduced LVEF of 35 % with the normal function of the prosthetic mitral valve. Laboratory tests suggested significantly elevated IL-6 of 91.3 pg/ml. Limitation of fluid, furosemide, dopamine, and dapagliflozin were prescribed to improve the cardiac output. From day 12 to day 13, levosimendan was prescribed for continuous intravenous infusion at the speed of 0.14μg/kg per minute for 24 hours, and synchronous cardiac telemetry showed that the patient was in sinus rhythm with occasional premature ventricular beats and heart rate ranging from 79/min to 95/min during the infusion. On day 13, the patient developed a sudden loss of consciousness with hemodynamic instability that lasted for 50 seconds. ECG monitoring showed an R-on-T event-initiated episode of TdP deteriorated into ventricular fibrillation (shown in Fig. 2a) and then converted to Af with a QTc of 454 ms under a heart rate of 140/min rapidly with chest compressions (shown in Fig. 2b). Lab work showed normal serum electrolytes (Na, K, Ca, Cl), arterial blood gas analysis, and a mild elevated troponin T and troponin I, 0.417 ng/ml and 0.587 ng/ml, respectively due to cardiac surgery. Craniocerebral computed tomography showed no abnormality compared with the previous image. The bedside echocardiogram showed no significant change from the previous. Five hours after the first TdP, cardiac arrest due to ventricular tachycardia and ventricular fibrillation appeared, and the patient was resuscitated with electric defibrillation, epinephrine, dopamine, lidocaine, chest compression, and urgent trachea intubation. She was transferred to the surgical intensive care unit. Upon arrival, the patient was in subcoma. Vital signs showed a blood pressure of 58/25 mmHg with continuous intravenous pumping of epinephrine (0.05 μg/kg per minute), dopamine (5 μg/kg per minute), and norepinephrine (0.04 μg/kg per minute), an irregular heart rate of 198 beats per minute with continuous infusion of lidocaine, oxygen saturation levels varying between 98 and 100 % with mechanical ventilation, and a temperature of 36.2 °C. Asynchronous electrical cardioversion, with the energy of 50J and 70J, respectively, was conducted because of tachycardia and severe hypotension, then atrioventricular node rhythm was restored and a prolonged QTc of 532 ms was shown on ECG. Repeated echocardiogram revealed reduced LVEF of 30 % with the normal function of the mechanical mitral valve, a newly emerged weakened motion of the left ventricle apex and middle segment, and normal movement of the basal segment which indicated the possibility of Takotsubo cardiomyopathy without left ventricular outflow tract obstruction so epinephrin was stopped. Urgent coronary angiography proved normal blood flow of coronaries without stenosis, and a temporary cardiac pacemaker via the right femoral vein was implanted at the same time to ensure the patient's safety in case the epicardial temporary pacing lost efficacy. On day 14, the patient's condition was still unstable with intravenous pumping of dopamine, norepinephrine, and lidocaine; T-wave alternation can be observed (shown in Fig. 3) followed by VT recurred including episodes of torsades de pointes (shown in Fig. 4) and epicardial temporary pacing was used with the rate of 90/min to suppress the ventricular arrhythmias effectively besides intravenous infusion of magnesium sulfate to maintain the serum magnesium in high normal levels (0.66–1.07mmol/L). Any medication that may cause a prolonged QT interval was prohibited. From day 15 to day 17, the patient had no further ventricular arrhythmia events with cardiac pacing. On day 17, lidocaine and norepinephrine were taken off, and atenolol was added at the dose of 6.25mg twice a day, which was withdrawn on day 18 because of severe bradycardia. On day 19, digoxin was prescribed at the dose of 0.125mg once a day, dopamine was reduced from 5ug/(kg. min) to 3ug/(kg. min) because of symptom relief and blood pressure in the normal range, and discontinued on day 20. On day 29, the follow-up echocardiography suggested that LVEDD reduced to 48mm and LVEF elevated to 45 %. Given the concern for genetic susceptibility to drug-induced long QT syndrome (LQTS), the patient's peripheral blood genomic DNA was sequenced, and positive pathogenic variant KCNQ1 (c.642C > T) was found, which had definitive evidence as a genetic cause of typical long QT syndrome type 1 (LQT1). From day 35, bradycardia occurred while the patient was taking digoxin, which was stopped immediately. On day 41, a prolonged QTc interval of 516 ms under a heart rate of 56 bpm was observed on ECG, and concern for ongoing TdP and cardiac arrest risk, a dual chamber implantable cardioverter defibrillator (Medtronic DDBC3D4) was placed on day 43. The patient was discharged from the hospital on day 46 with the prescription of potassium magnesium aspartate tablets, warfarin, dapagliflozin, furosemide, and spironolactone (Table 1).

**Table 1:** Time line.

Date		Clinical events	QTc (ms)	IL-6 (0–7pg/ml)	NT-proBNP (0–300 pg/ml)	Medications
1		admission (Af)	435	5.4	2709	DIG, furosemide, spironolactone, sacubitril/valsartan, dapagliflozin
8		surgery	448	–	–	furosemide, KCl
9		paroxysmal Af	452	91.3	2621	warfarin, furosemide, KCl
10		sinus	441	149.9	4525	DA, warfarin, furosemide, KCl, dapagliflozin
12 10:01		sinus	–	–	–	start of levosimendan infusion, DA, furosemide, KCl, warfarin
13	10:05	PVC	–	–	–	end of levosimendan infusion, DA, furosemide, KCl
	11:07	first TdP	454	–	–	DA, KCl
	18:21	VT, VF	–	–	–	DA, lidocaine, KCl
	18:29	CPR	–	–	–	E, DA, lidocaine
	18:50	AV node rhythm	532	–	–	E, DA, NE, lidocaine, MgSO_4_
	20:02	Takotsubo cardiomyopathy	495	–	–	E, DA, NE, lidocaine, MgSO_4_
14		T-wave alternation,TdP	453	41.9	6918	DA, NE, lidocaine, MgSO_4,_ warfarin
15		VPR	–	59.6	8979	DA, NE, lidocaine, warfarin
16		VPR	–	38.6	1754	DA, NE, lidocaine, warfarin
17		VPR	–	27.0	1438	Atenolol, DA, warfarin
18		VPR	–	9.0	623	DA, furosemide, KCl,warfarin
19		Af	–	5.2	495	DIG, DA,warfarin
25		Af	437	–	–	DIG,warfarin
35		Af	–	–	–	warfarin
41		VPR	516	–	–	warfarin
43		ICD (atrial pacing)	473	–	–	warfarin
46		Discharge (atrial pacing)	–	–	–	potassium magnesium aspartate, dapagliflozin, furosemide,spironolacton, warfarin

Af: atrial fibrillation; QTc: QT intervals corrected for cardiac frequency using Fridericia's formula; IL-6: Interleukin-6; NT-proBNP: N-terminal B-type brain natriuretic peptide precursor; Af: atrial fibrillation; DIG: digoxin; KCl: potassium chloride; PVC: premature ventricular contraction; DA: dopamin; TdP: Torsades de points; VT: ventricular tachycardia; VF: ventricular fibrillation; CPR: cardiopulmonary resuscitation; E: epinephrin; NE: norepinephrin; AV node: atrioventricular node; MgSO_4_: magnesium sulfate; VPR: ventricular pacing rhythym; ICD: implantable cardioverter defibrillator.

The tangent method was used to define the end of the T-wave in the lead with the longest QT interval. All measured QT intervals were corrected for cardiac frequency using Fridericia's formula [10,11]. Prolongation of the QT interval was defined as a QTc interval ≥460 ms in females [12].

## 2 Discussion

Levosimendan is a widely used calcium sensitizer in acute decompensated heart failure without increased cardiac oxygen consumption. Here we describe a rare case with several episodes of TdP and cardiac arrest in a complicated clinical setting of levosimendan administration post-cardiac operation with underlying LQT1.

Congenital LQTS has been one of the most investigated cardiac ion channel diseases, which can affect certain ion currents and then increase the ventricular action potential duration inhomogeneously and hence the QT interval that provides the substrate of ventricular tachycardia associated with LQTS, commonly referred to as TdP [13]. According to the molecular genetics study, LQT1 is one of the most common subtypes of congenital LQTS caused by the loss-of-function mutations on KCNQ1 [1]. Arrhythmia events, typically manifested as TdP, can be triggered by different inducements, for example, exercise, stress, and sudden auditory stimuli, for different genotypes of congenital LQTS, and exercise is the most frequent trigger for LQT1 [1]. However, congenital LQTS is relatively rare, as reported. Conversely, acquired LQTS is rather common, most frequently associated with electrolyte imbalances, medications, heart failure, structural heart diseases, and, more recently, inflammation and autoimmunity [[1], [2], [3], [4]]. Some specific medications account for a substantial part of those [14]. Considerable drugs, including widely used antibiotics, antidepressants, and cardiovascular drugs, have been reported before, which are closely linked with QT interval prolongation and TdP [15], but TdP potentially associated with levosimendan has not received widespread attention yet.

Sun Yue et al. first reported that a 72-year-old female who was diagnosed with dilated cardiomyopathy and heart failure developed TdP and Aspen syndrome during the use of levosimendan (continuous intravenous pumping at a speed of 6.25μg per minute for 20 hours, patient's weight was unknown) along with hypokalemia. However, the case failed to do the DNA test [16]. Our case was a postoperative female who received continuous intravenous pumping of levosimendan at the speed of 0.14μg/kg per minute (recommended dose 0.05–0.2 μg/kg per minute) and lasted for 24 hours without electrolyte imbalance. Both cases denied prior severe arrhythmia events, and the initial QTc interval was normal. The therapeutic concentration of levosimendan is reached about 1 hour after the start of intravenous infusion, and a steady state is reached within 5 hours after continuous infusion initiation [17]. After a 24-h infusion of levosimendan, the pharmacodynamic effect of the drug is observed for at least one week [17]. The proarrhythmic effects of levosimendan are still controversial. Landoni et al. concluded that there was no significant difference between the levosimendan group and the placebo group in rates of cardiac arrhythmias with patients in whom perioperative hemodynamic support was indicated after cardiac surgery [18]. Few studies, including the randomized, placebo-controlled LEAF-trial, have reached the same conclusion [19]. Levosimendan has not yet appeared on the most recent CredibleMeds list of accepted QT prolongators. However, a potential association with ventricular arrhythmias and deaths from levosimendan has been observed in clinical studies [9,20]. Limited studies demonstrated that intravenous levosimendan increases the heart rate and prolongs QTc interval in healthy volunteers and patients with chronic heart failure [9]. Moreover, the magnitude of such increases is critically dose-dependent, and these increases are small at therapeutically relevant doses and plasma drug levels [9]. Furthermore, in vitro study has shown that acute infusion of levosimendan may lead to an increased occurrence of ventricular tachyarrhythmias in an experimental whole-heart model and the underlying mechanism for the increased inducibility of ventricular fibrillation in the present study is a significant abbreviation of ventricular repolarization and a reduction of ventricular refractory period [21].

Considering the complex clinical setting, it is difficult to be assured that the TdP in our case is caused by levosimendan alone. According to the current literature, in most cases, multiple risk factors need to be simultaneously present to develop QTc prolongation because numerous often‐redundant ion channel mechanisms are implicated in preserving the normal action potential, multiple QT‐prolonging factors are required to disrupt ventricular repolarization significantly [22]. In addition to the underlying congenital LQTS, possible acquired risk factors for TdP were excluded as possible in the case, for example, electrolyte disturbance, potential drugs to prolong QT interval like amiodarone, mechanical valvular dysfunction, coronary stenosis, hypothyroidism, hypothermia, and autoimmune disorder. However, according to the patient's clinical course and current literature, other acquired factors except levosimendan administration may have been involved in determining the occurrence of the TdP, such as the use of epinephrine [23] and possible Takotsubo syndrome [24]. Ventricular arrhythmias, including TdP, are described in up to 3–20 % of patients with Takotsubo syndrome [[24], [25], [26], [27]]. Even though levosimendan seems to accelerate recovery in patients with Takotsubo cardiomyopathy [28], the occurrence of TdP during treatment has nevertheless been described [29]. Furthermore, Takotsubo patients typically show increased circulating inflammatory markers, such as C-reactive protein (CRP), and IL-6, correlated with reduced left ventricular ejection fraction, and increased arrhythmic events and mortality [30,31]. As reported in our case, the patient's laboratory examination revealed a significantly elevated IL-6, consistent with recent literature. Indeed, inflammation has recently been shown to prolong ventricular action potential duration, and a role for Il-6 has been demonstrated [32]. Inflammation is now considered a non-conventional risk factor for long QTc [32]. Thus, the possibility that Takotsubo syndrome, with the contribution of an underlying inflammatory state, possibly in co-operation with the concomitant levosimendan infusion, may have contributed to the arrhythmic storm.

In conclusion, according to the WHO-UMC system for standardized case causality assessment, the causal relationship between TdP and levosimendan is adjudicated as ‘possible’ considering the underlying congenital LQTS, epinephrin administration, possible Takotsubo syndrome with inflammation and heart failure in our case [33].

There are some limitations to our study. We failed to test the levosimendan plasma drug concentration given the lack of proper testing conditions and the female's normal hepatic and renal function, which may not affect the metabolism of levosimendan. Besides, more relevant research on levosimendan in long QT syndrome needs to be conducted. In addition, cardiac telemetry records were not retained for some of the critical time points in the patient's hospitalization.

This case report indicates that levosimendan, a known ‘safe’ drug, may have the potential to cause life-threatening arrhythmias in patients with congenital long QT syndrome in complex clinical situations. In addition, this case is a good opportunity to remind the cardiologists. There should be a careful evaluation of the at-risk populations and close monitoring of the electrocardiograms, particularly the QT interval, to reduce the risk of near-fatal arrhythmias during the use of levosimendan.

## 3 Conclusion

In conclusion, we describe a near-fatal postoperative case of TdP and cardiac arrest requiring advanced cardiac life support followed by implantation of a cardioverter defibrillator. Concomitant levosimendan infusion with underlying congenital LQTS, along with epinephrin administration, possible Takotsubo syndrome with inflammation and heart failure, may have contributed to it. The possibility of TdP as a severe complication of levosimendan compatible with congenital LQTS in complex clinical situations should be remembered.

## Informed consent statement

Written informed consent was obtained from the patient for publication of this case report.

## Ethics declarations

This study was reviewed and approved by Fuwai Hospital Chinese Academy of Medical Sciences, Shenzhen Ethics Committee, with the approval number: SP2022034(01).

The patient (or their proxies/legal guardians) provided informed consent to participate in the study and the publication of their anonymised case details and images.

## Funding statement

This work was supported by Shenzhen Key Medical Discipline Construction Fund (No. SZXK080), Shenzhen Key Medical Discipline Construction Fund (No. SZXK019), Shenzhen High-level Hospital Construction Fund and Young Talent Program of the Academician Fund (No.YS-2020-013).

## Data availability statement

The data associated with our study has not been deposited into a publicly available repository yet and the contributions presented in the study are included in the article, further inquiries can be directed to the corresponding author.

## CRediT authorship contribution statement

**Fengyan Zha:** Writing – original draft, Investigation, Formal analysis, Data curation, Conceptualization. **Xing Li:** Investigation, Formal analysis, Data curation, Conceptualization. **Hui Yin:** Investigation, Formal analysis, Data curation, Conceptualization. **Di Huang:** Data curation. **Yu Du:** Writing – review & editing, Conceptualization. **Chuzhi Zhou:** Writing – review & editing, Investigation, Formal analysis, Data curation, Conceptualization.

## Declaration of competing interest

The authors declare that they have no known competing financial interests or personal relationships that could have appeared to influence the work reported in this paper.
